# Integrated Transcriptomic and Metabolomic Analysis Reveals MeJA-Mediated Flavonoid Biosynthesis in *Cyclocarya paliurus* Callus

**DOI:** 10.3390/plants15203135

**Published:** 2026-10-14

**Authors:** Yilin Gao, Kaidi Gao, Yiting Wang, Zhuosui Li, Jian Qin, Xulan Shang

**Affiliations:** 1 State Key Laboratory for the Development and Utilization of Forest Food Resources, Nanjing Forestry University, Nanjing 210037, China; 13776082149@163.com (Y.G.); m15938713715@163.com (K.G.); 18771492998@163.com (Y.W.); years616@163.com (Z.L.); 2 College of Forestry and Grassland, Nanjing Forestry University, Nanjing 210037, China; 3 Co-Innovation Center for the Sustainable Forestry in Southern China, Nanjing Forestry University, Nanjing 210037, China; 4 Institute of Fruit Tree Research, Guangdong Academy of Agricultural Sciences, Key Laboratory of South Subtropical Fruit Biology and Genetic Resource Utilization, Ministry of Agriculture and Rural Affairs, Guangdong Provincial Key Laboratory of Science and Technology Research on Fruit Tree, Guangzhou 510640, China; qinjian320@163.com

**Keywords:** *Cyclocarya paliurus*, methyl jasmonate, flavonoid biosynthesis, transcriptomics, metabolomics

## Abstract

*Cyclocarya paliurus* is a rare Chinese endemic medicinal tree with high-value bioactive metabolites in its leaves, but wild resource scarcity and growth constraints limit large-scale utilization. Callus culture combined with methyl jasmonate (MeJA) elicitation is a promising strategy for sustainable bioactive compound production. However, the metabolic responses of *C. paliurus* callus to MeJA and the underlying molecular regulatory mechanisms remain unclear. Here, in vitro *C. paliurus* callus treated with gradient concentrations of MeJA was analyzed by integrating untargeted metabolomics and transcriptomics. Exogenous MeJA significantly induced the accumulation of major flavonoids in *C. paliurus* callus, including dihydrokaempferol, dihydroquercetin, and quercetin. Twelve key structural genes involved in flavonoid biosynthesis (two *4CL* genes, two *CHS* genes, one *CYP73A* gene, three *HCT* genes, one *CCoAOMT* gene, one *CHR* gene, one *CYP75A* gene, and one *ANS* gene) were co-upregulated. Two hub transcription factors (TFs), *CpbHLH162* and *CpNAC073*, were identified as potential upstream regulators of flavonoid synthesis. These findings indicate that MeJA enhances flavonoid production in *C. paliurus* callus by activating multiple structural genes and key transcription factors, providing candidate genes for metabolic improvement and theoretical support for the large-scale industrial production of bioactive compounds in *C. paliurus*.

## 1. Introduction

*Cyclocarya paliurus* (Batalin) Iljinsk., a rare medicinal tree belonging to the Juglandaceae family, is an endemic species unique to China [1,2]. Its leaves accumulate abundant high-value bioactive compounds, mainly including flavonoids, triterpenoids, and polysaccharides. These functional components endow *C. paliurus* with multiple pharmacological properties, such as antihypertensive, hypoglycemic, hypolipidemic, and anti-tumor activities [3,4,5,6]. As the core chemical basis of the medicinal efficacy of *C. paliurus*, the composition and accumulation abundance of these bioactive compounds are critical indicators for evaluating the comprehensive quality of this medicinal plant.

Owing to its excellent medicinal and healthcare functions, *C. paliurus* possesses broad prospects for comprehensive development and industrial utilization. Nevertheless, the large-scale application of wild *C. paliurus* is greatly limited by scarce natural resources, long growth cycles, as well as seasonal and geographical constraints [7]. Plant callus culture serves as an effective alternative to address these bottlenecks. This in vitro cultivation system has the advantages of a short growth cycle, controllable culture conditions, and freedom from seasonal and geographical restrictions [8], and has been successfully applied for the sustainable biosynthesis of bioactive compounds in medicinal plants, as reported in *Bacopa monnieri* and *Mentha longifolia* [9,10].

Methyl jasmonate (MeJA) acts as a key exogenous elicitor that effectively promotes metabolite accumulation in plants [11,12]. It can simulate plant endogenous stress signals and trigger the transcriptional regulation of secondary metabolic synthesis genes through activating transcription factors (TFs), thereby facilitating the accumulation of bioactive compounds in medicinal plants [13,14,15]. Previous studies have shown that MeJA-mediated regulatory patterns of synthetic genes exhibit significant species-specificity. For instance, MeJA induced *GiMYB76,* which directly bound to the *GiCHS4* promoter and activated its expression, thereby promoting licochalcone A accumulation in *Glycyrrhiza inflata* [16], whereas, in *Aralia elata*, it preferentially up-regulated the triterpenoid biosynthetic gene *Aeβ-AS* through *AeMYB9* and *AeMYB73*, resulting in a marked increase in oleanolic acid and hederagenin content [17]. In addition, the regulatory mechanism of MeJA on secondary metabolism in callus has also been reported in recent years, such as *Eriobotrya japonica* [18], *Pyrus sinkiangensis* [19] and *Damnacanthus major* [20].

However, in *C. paliurus*, existing studies have only demonstrated that MeJA promotes triterpenoid accumulation in leaves, while systematic dissection at the global secondary metabolism level and the underlying molecular regulatory mechanisms remain largely unexplored [21]. Therefore, in vitro-cultured *C. paliurus* callus was treated with different concentrations of MeJA in this study. By integrating untargeted metabolomics and transcriptomics, this study aims to systematically investigate the effects of MeJA on the metabolic response characteristics of bioactive components and the underlying transcriptional regulatory mechanisms in *C. paliurus* callus. The findings of this study provide systematic omics evidence for revealing the molecular mechanism by which MeJA regulates secondary metabolism through modulating synthetic gene expression in *C. paliurus*, and offer a reliable theoretical basis and technical reference for the exogenous targeted regulation and large-scale industrial production of high-value bioactive compounds in this medicinal plant.

## 2. Results

### 2.1. Effects of MeJA on Growth in C. paliurus Callus

**Figure 1:**
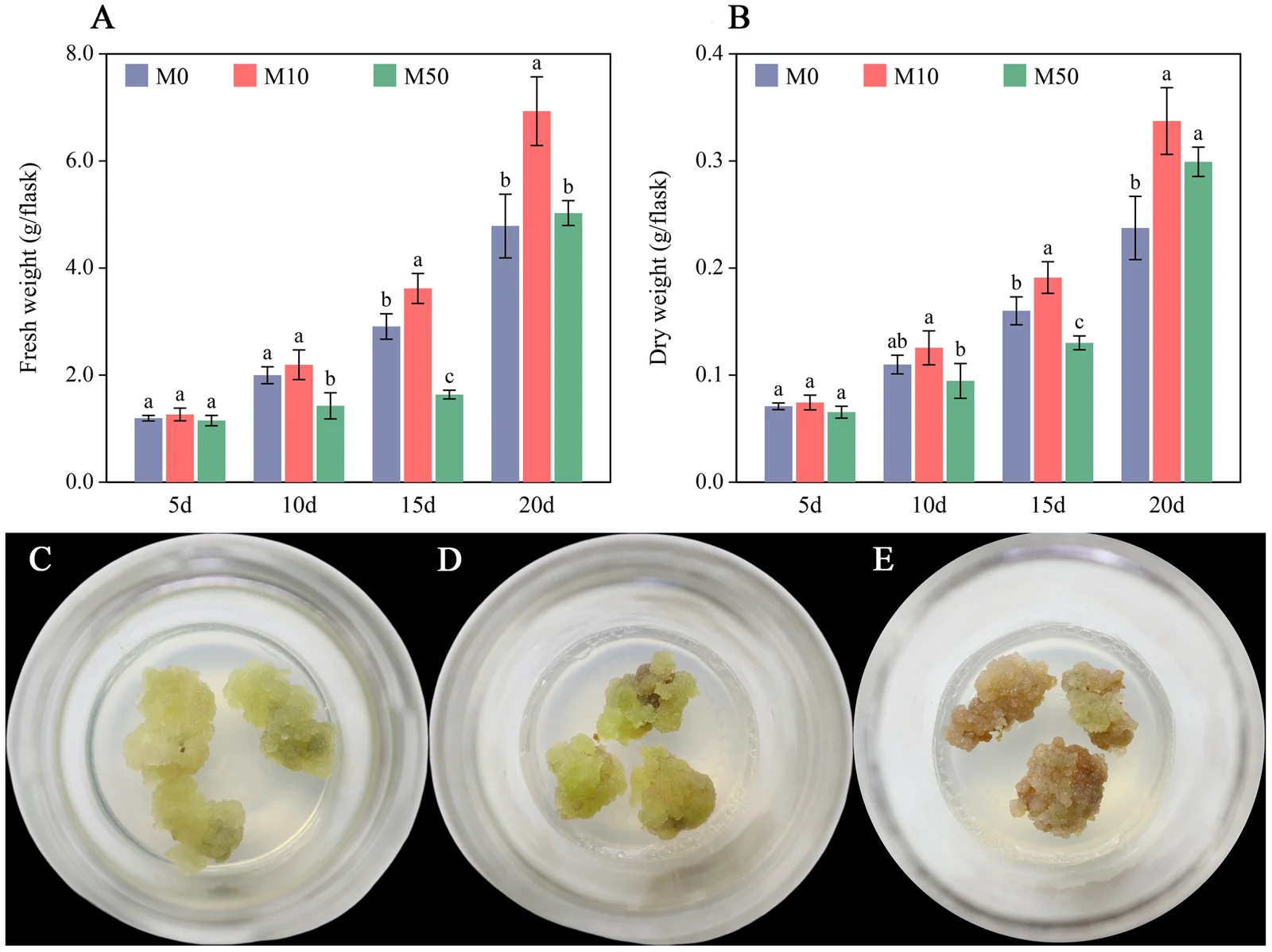
Effects of MeJA on callus growth in *C. paliurus* callus. (**A**) Fresh weight. (**B**) Dry weight. (**C**) Color of *C. paliurus* callus under the M0 treatment after 20 days of culture. (**D**) Color of *C. paliurus* callus under the M10 treatment after 20 days of culture. (**E**) Color of *C. paliurus* callus under the M50 treatment after 20 days of culture. Note: Different lowercase letters in the figure indicate significant differences among treatments (*p <* 0.05).

As shown in Figure 1A,B and Table S1, the fresh weight and dry weight of *C. paliurus* callus increased with culture time in all treatments. Significant differences in fresh weight were detected among MeJA concentrations at all time points except 5 days, whereas for dry weight significant differences were observed at 15 and 20 days but not at 5 or 10 days (*p* < 0.05). At 20 days, the highest fresh weight was recorded under the M10 treatment (6.93 g/flask), which was significantly higher than that of the other treatments; the corresponding dry weight was 0.34 g/flask, which did not differ significantly from that under the M50 treatment but was significantly higher than that of the control. In addition, at 20 days, the callus color gradually changed from light green to reddish-brown as the MeJA concentration increased (Figure 1C–E).

### 2.2. Screening of Differentially Accumulated Metabolites

**Figure 2:**
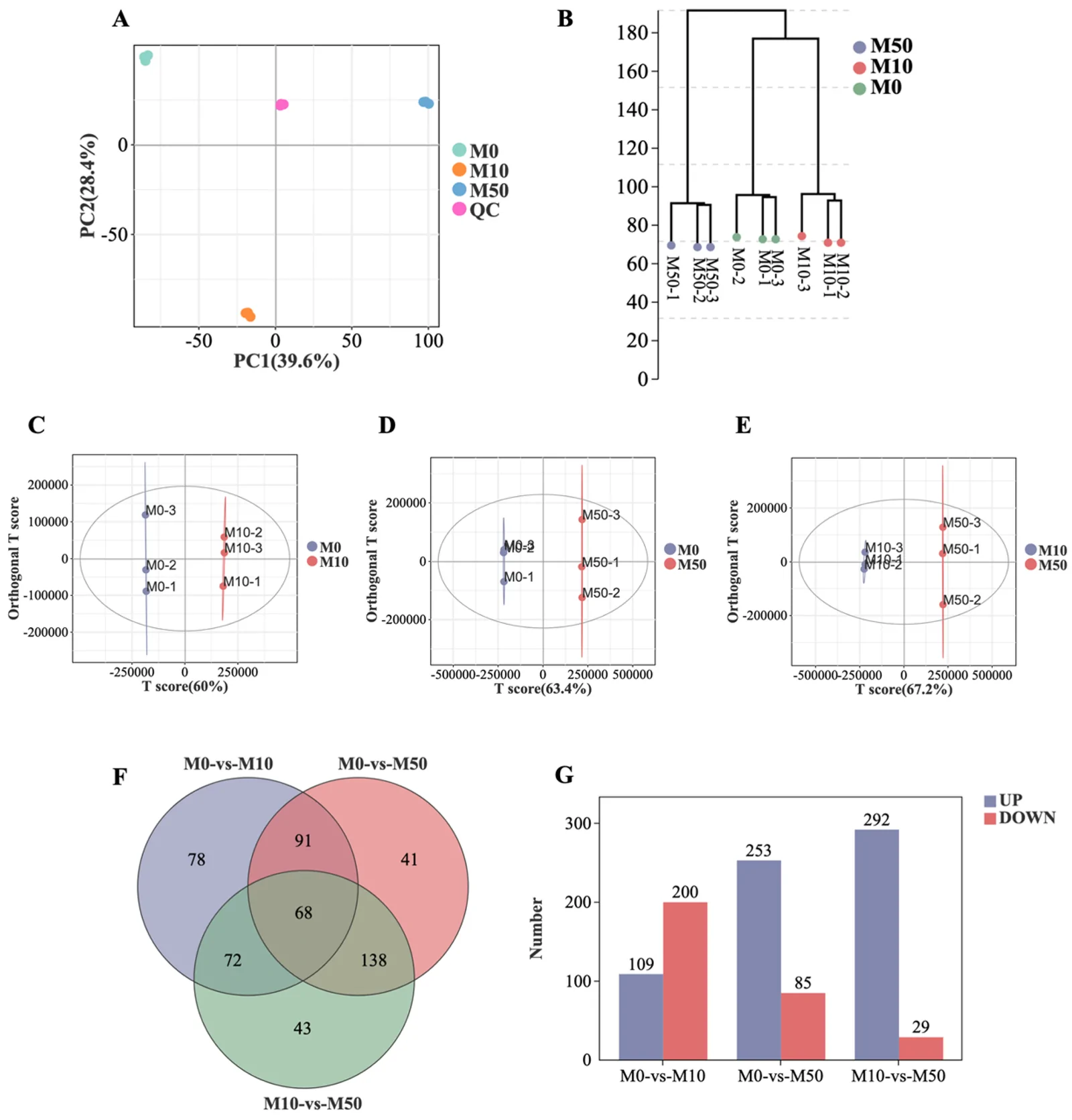
Screening of differentially accumulated metabolites. (**A**) Principal component analysis; QC, quality control samples. (**B**) Cluster analysis. (**C**) OPLS-DA between M0 and M10. (**D**) OPLS-DA between M0 and M50. (**E**) OPLS-DA between M10 and M50. (**F**) Venn diagram of differentially accumulated metabolites among comparison groups. (**G**) Number of differentially accumulated metabolites identified in each group.

To investigate the metabolic responses of *C. paliurus* callus to different concentrations of MeJA, untargeted metabolomic analysis was performed using ultra-high performance liquid chromatography coupled with tandem mass spectrometry (UHPLC-MS/MS). Principal component analysis (PCA) (Figure 2A) showed that the three replicates of each treatment tended to cluster together, while clear separation was observed among the different treatments. The first principal component (PC1) and the second principal component (PC2) explained 39.6% and 28.4% of the total variance, respectively. Cluster analysis further supported these findings (Figure 2B).

Subsequently, an orthogonal partial least-squares discriminant analysis (OPLS-DA) model was constructed for the M0 vs. M10, M0 vs. M50, and M10 vs. M50 comparisons. As shown in Figure 2C–E, the first predictive component (T Score) explained 60.0%, 63.4%, and 67.2% of the total variance for the three comparisons, respectively. All samples were located within the 95% confidence ellipses (Hotelling’s T^2^) of their respective groups, and no outliers were detected, indicating good intra-group repeatability. Model validation showed that the cross-validated Q^2^ values were 0.985, 0.987, and 0.990 for the three comparisons, respectively, all exceeding 0.900. These results indicated that the models had high predictive ability under cross-validation and that the identified differentially accumulated metabolites (DAMs) were representative.

According to the Venn diagram of DAMs in the M0 vs. M10, M0 vs. M50, and M10 vs. M50 comparisons, a total of 531 DAMs were identified (Figure 2F and Table S2). Among them, 68 DAMs were shared among the three comparisons, while 78, 41, and 43 DAMs were unique to each comparison, respectively. As shown in Figure 2G, the numbers of DAMs in the three comparisons were 309 (109 up, 200 down), 338 (253 up, 85 down), and 321 (292 up, 29 down), respectively.

### 2.3. MeJA-Induced Flavonoid Accumulation

**Figure 3:**
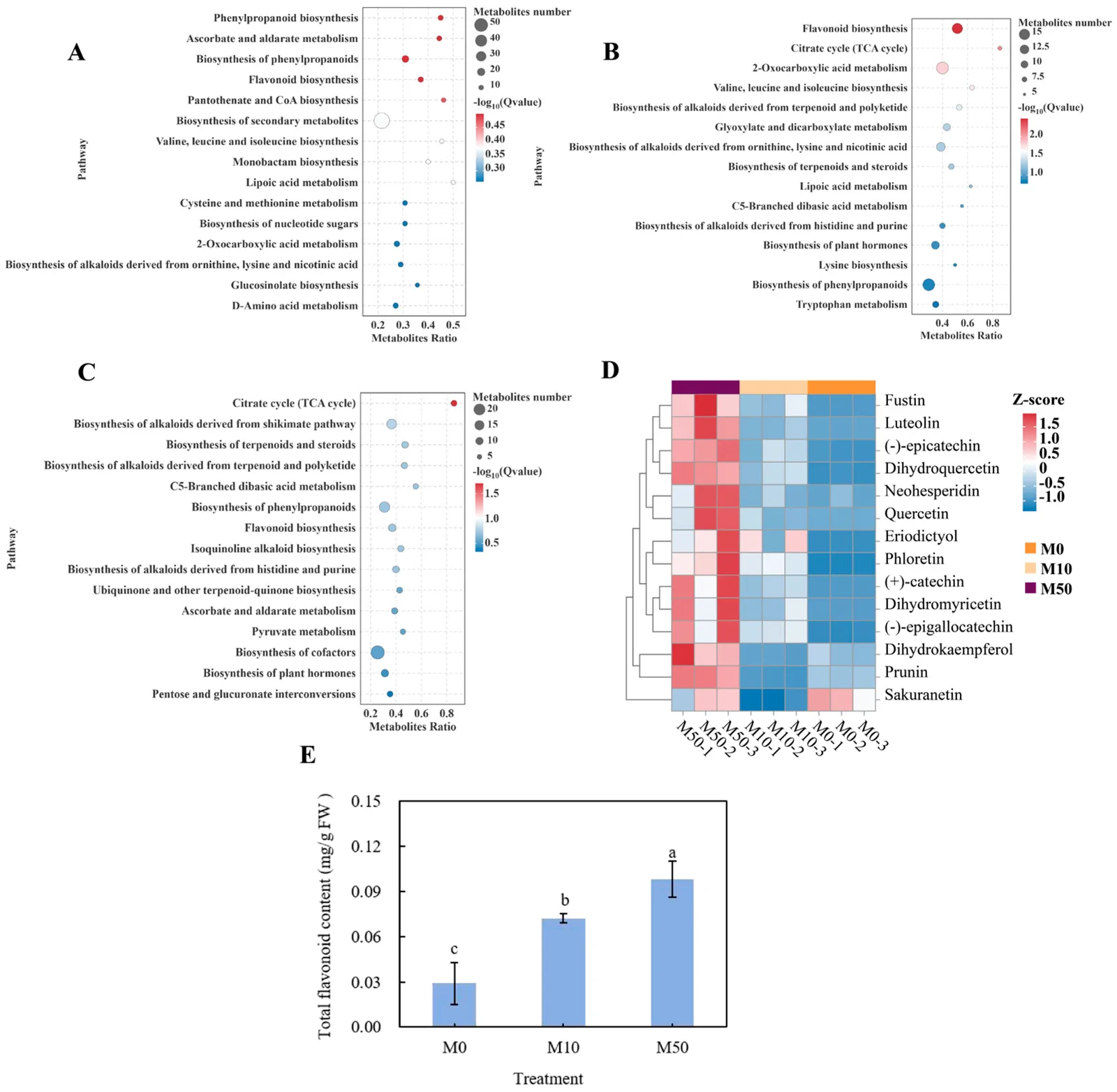
Analysis of flavonoid-related differentially accumulated metabolites. (**A**) KEGG enrichment analysis of differentially accumulated metabolites between M0 and M10. (**B**) KEGG enrichment analysis of differentially accumulated metabolites between M0 and M50. (**C**) KEGG enrichment analysis of differentially accumulated metabolites between M10 and M50. (**D**) Cluster heatmap of flavonoid-related differentially accumulated metabolites. (**E**) Total flavonoid content. Note: Different lowercase letters in the figure indicate significant differences among treatments (*p <* 0.05).

To further explore the biological functions of the DAMs, KEGG enrichment analysis was performed. For each comparison group, the 15 pathways with the lowest false discovery rate (FDR) values were selected (Figure 3A–C). The flavonoid biosynthesis pathway was enriched in all three comparisons.

As shown in Figure 3D, 14DAMs were annotated to the flavonoid biosynthesis pathway, including dihydroquercetin, dihydrokaempferol, fustin, (+)-catechin, (-)-epicatechin, eriodictyol, dihydromyricetin, (-)-epigallocatechin, luteolin, quercetin, neohesperidin, phloretin, prunin, and sakuranetin. Previous studies have reported that flavonoids in *C. paliurus* are dominated by kaempferol-type and quercetin-type compounds [22]. Among the DAMs identified in this study, these two types include dihydrokaempferol, dihydroquercetin and quercetin. The relative contents of dihydrokaempferol, dihydroquercetin, and quercetin each increased progressively with increasing MeJA concentration.

Significant differences in total flavonoid content were observed among treatments (Figure 3E; *p* < 0.05). Total flavonoid content increased progressively with increasing MeJA concentration and reached a maximum of 0.10 mg/g fresh weight under the M50 treatment, which was 3.38-fold that of M0. This result was consistent with the metabolomic data.

### 2.4. Screening of Differentially Expressed Genes

Transcriptomic sequencing was performed to reveal changes in gene expression after MeJA treatment. A total of 35.99–55.05 million raw reads were generated per sample, and 35.92–54.96 million clean reads were retained after quality filtering. The Q30 values and GC contents of all samples ranged from 95.48% to 96.19% and from 45.16% to 45.52%, respectively, indicating that the sequencing data were of high quality and suitable for subsequent analysis (Table S3).

**Figure 4:**
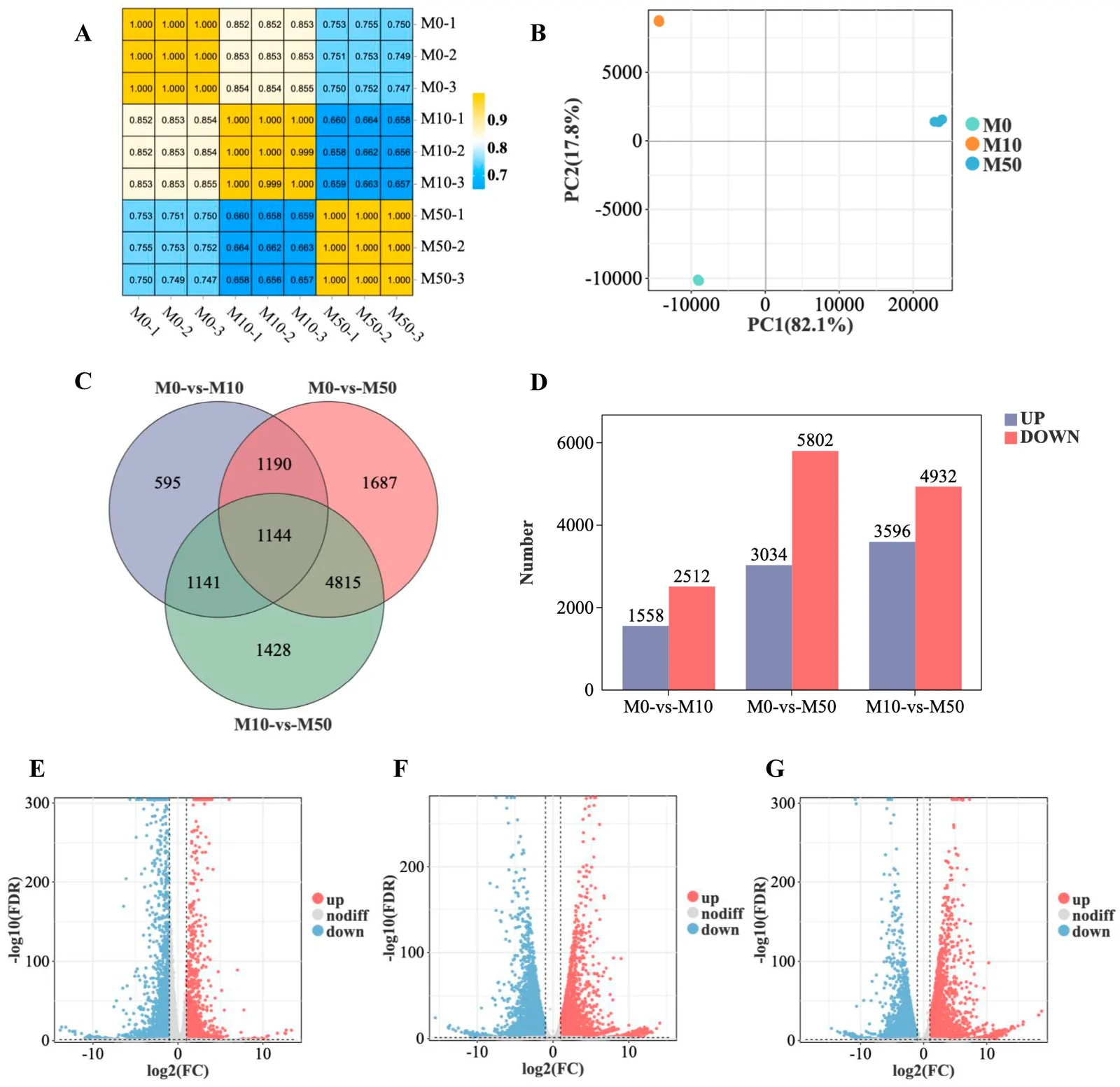
Screening of differentially expressed genes. (**A**) Pearson correlation analysis. (**B**) Principal component analysis. (**C**) Venn diagram of differentially expressed genes among comparison groups. (**D**) Number of differentially expressed genes identified in each comparison group. (**E**) Volcano plot of differentially expressed genes between M0 and M10. (**F**) Volcano plot of differentially expressed genes between M0 and M50. (**G**) Volcano plot of differentially expressed genes between M10 and M50.

Correlation of gene expression profiles between samples is widely used to evaluate the reliability of transcriptomic data. As shown in Figure 4A, the Pearson correlation coefficients among biological replicates were all greater than 0.990, indicating strong correlations and high reproducibility between replicates. PCA (Figure 4B) further supported these findings: the three replicates of each group tended to cluster together and the different groups were clearly separated. PC1 and PC2 explained 82.1% and 17.8% of the total variance, respectively.

According to the Venn diagram of differentially expressed genes (DEGs) in the M0 vs. M10, M0 vs. M50, and M10 vs. M50 comparisons, a total of 12,000 DEGs were identified (Figure 4C and Table S4). Among them, 1144 DEGs were shared by all three comparisons, while 595, 1687, and 1428 DEGs were unique to each comparison, respectively. As shown in Figure 4D–G, 4070 (1558 up and 2512 down), 8836 (3034 up and 5802 down), and 8528 (3596 up and 4932 down) DEGs were detected in the three comparisons, respectively.

### 2.5. Enrichment Analyses of Differentially Expressed Genes and Activation of the Flavonoid Biosynthesis Pathway

**Figure 5:**
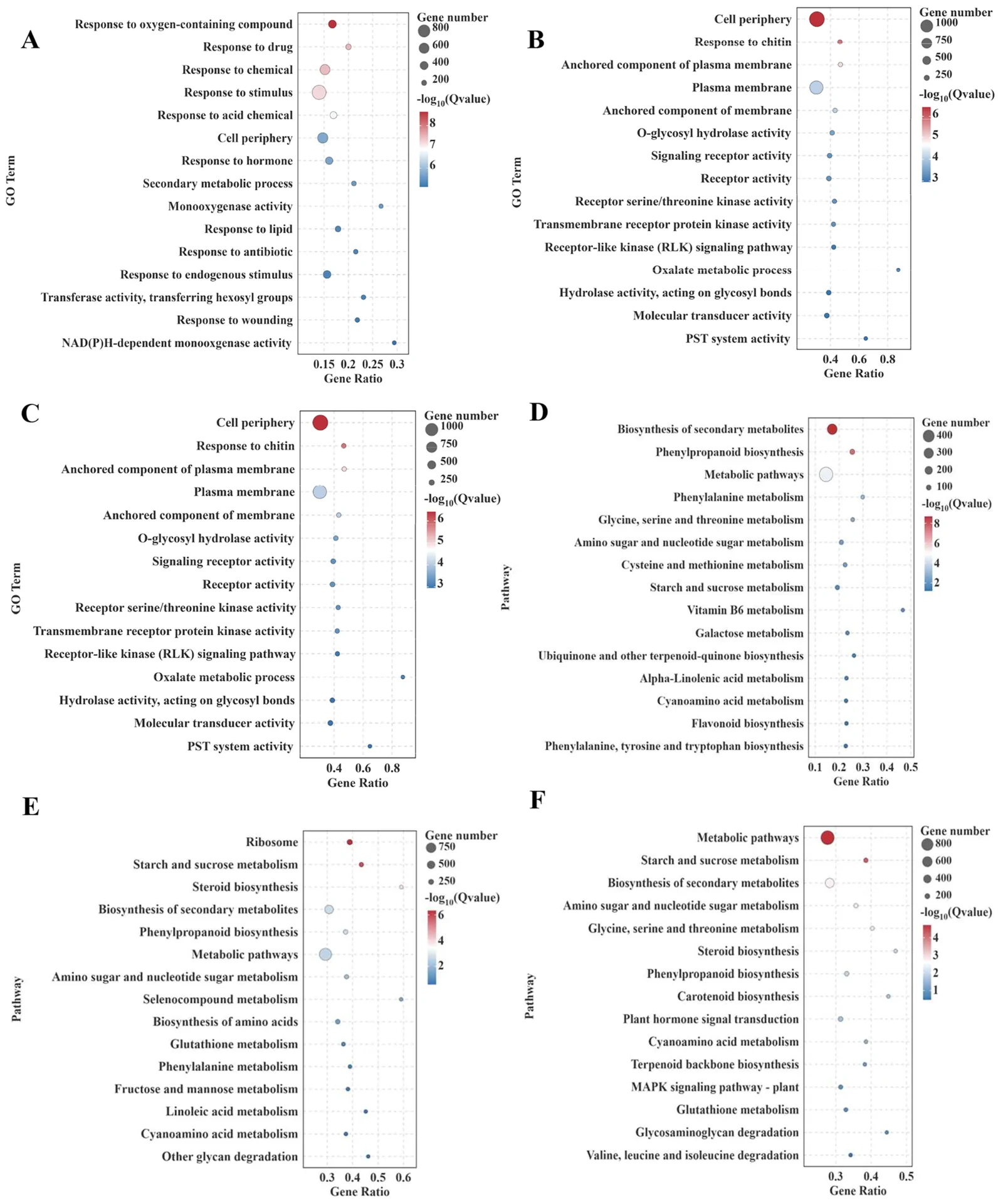
GO and KEGG enrichment analysis of differentially expressed genes. (**A**) GO enrichment analysis of differentially expressed genes between M0 and M10. (**B**) GO enrichment analysis of differentially expressed genes between M0 and M50. (**C**) GO enrichment analysis of differentially expressed genes between M10 and M50. (**D**) KEGG enrichment analysis of differentially expressed genes between M0 and M10. (**E**) KEGG enrichment analysis of differentially expressed genes between M0 and M50. (**F**) KEGG enrichment analysis of differentially expressed genes between M10 and M50.

To further assess the transcriptomic changes in *C. paliurus* callus under MeJA treatment and the potential functions of DEGs, GO and KEGG enrichment analyses were performed for each comparison. For GO enrichment, the top 15 GO terms with the smallest FDR values were selected for each comparison (Figure 5A–C). Among the M0 vs. M10, M0 vs. M50, and M10 vs. M50 comparisons, 11, 2, and 3 GO terms were enriched in biological processes, respectively; 3, 2, and 8 in molecular functions; and 1, 11, and 4 in cellular components, respectively. For KEGG enrichment, the 15 pathways with the smallest FDR values were selected for each comparison (Figure 5D–F), and pathways with FDR < 0.05 were defined as significantly enriched. A total of 13, 9, and 10 significantly enriched pathways were identified in the M0 vs. M10, M0 vs. M50, and M10 vs. M50 comparisons, respectively. Metabolic pathways, biosynthesis of secondary metabolites, and phenylpropanoid biosynthesis were enriched in all three comparisons.

**Figure 6:**
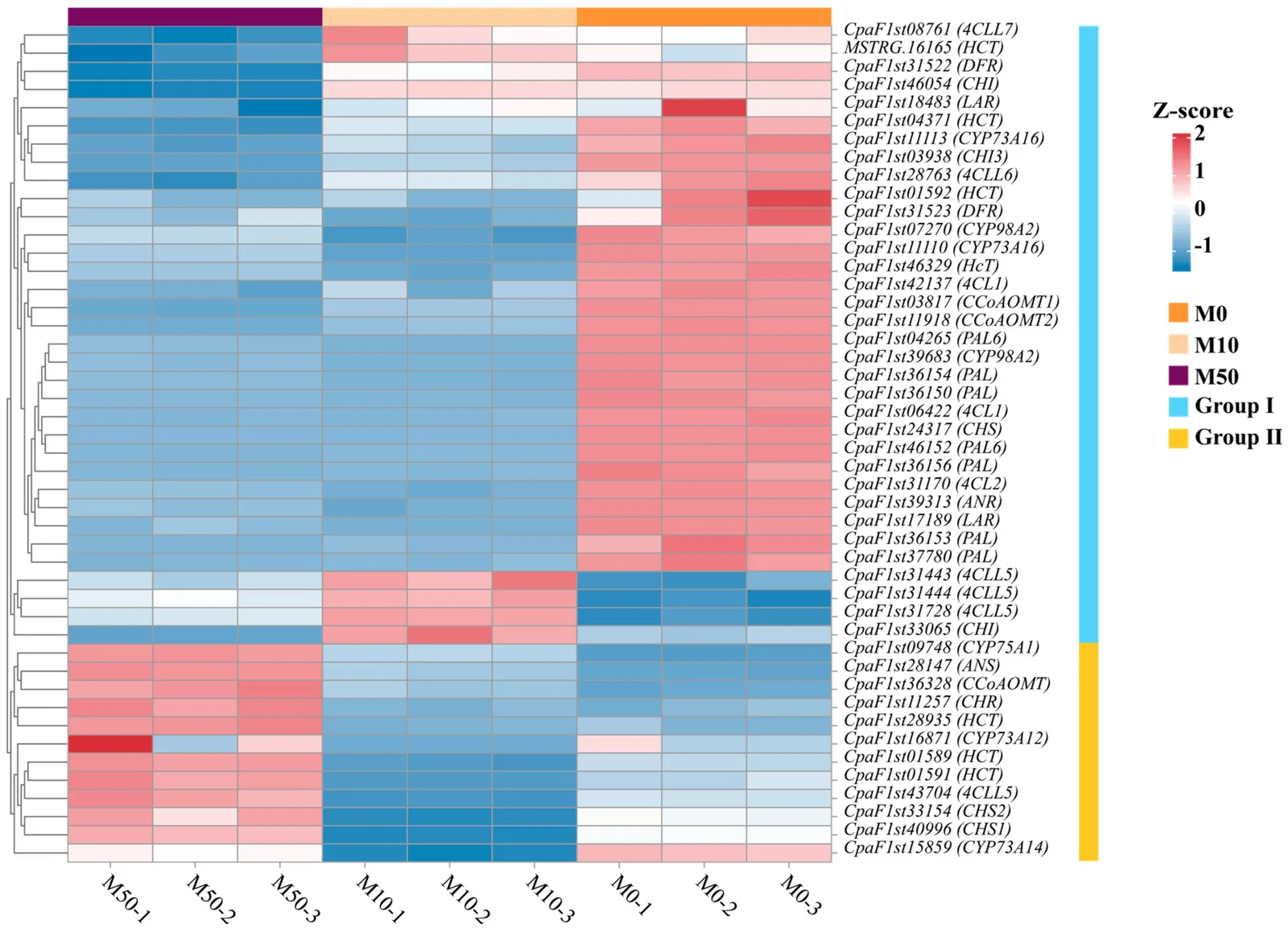
Clustering heatmap of flavonoid biosynthesis-related differentially expressed genes.

Based on the transcriptomic analysis, flavonoid biosynthesis-related DEGs were selected for hierarchical clustering analysis. A total of 46 DEGs were identified and classified into two groups according to their expression patterns across the M0, M10, and M50 treatments (Figure 6). As shown in Group I, the majority of genes exhibited high expression under M0, whereas a subset of genes reached high expression levels under M10. In contrast, 12 genes in Group II were highly expressed under M50 but showed low expression under M0 and M10, indicating that this set of structural genes is co-activated at the highest MeJA concentration. These expression patterns indicate that MeJA differentially regulates the expression of multiple flavonoid biosynthetic structural genes in a concentration-dependent manner.

**Figure 7:**
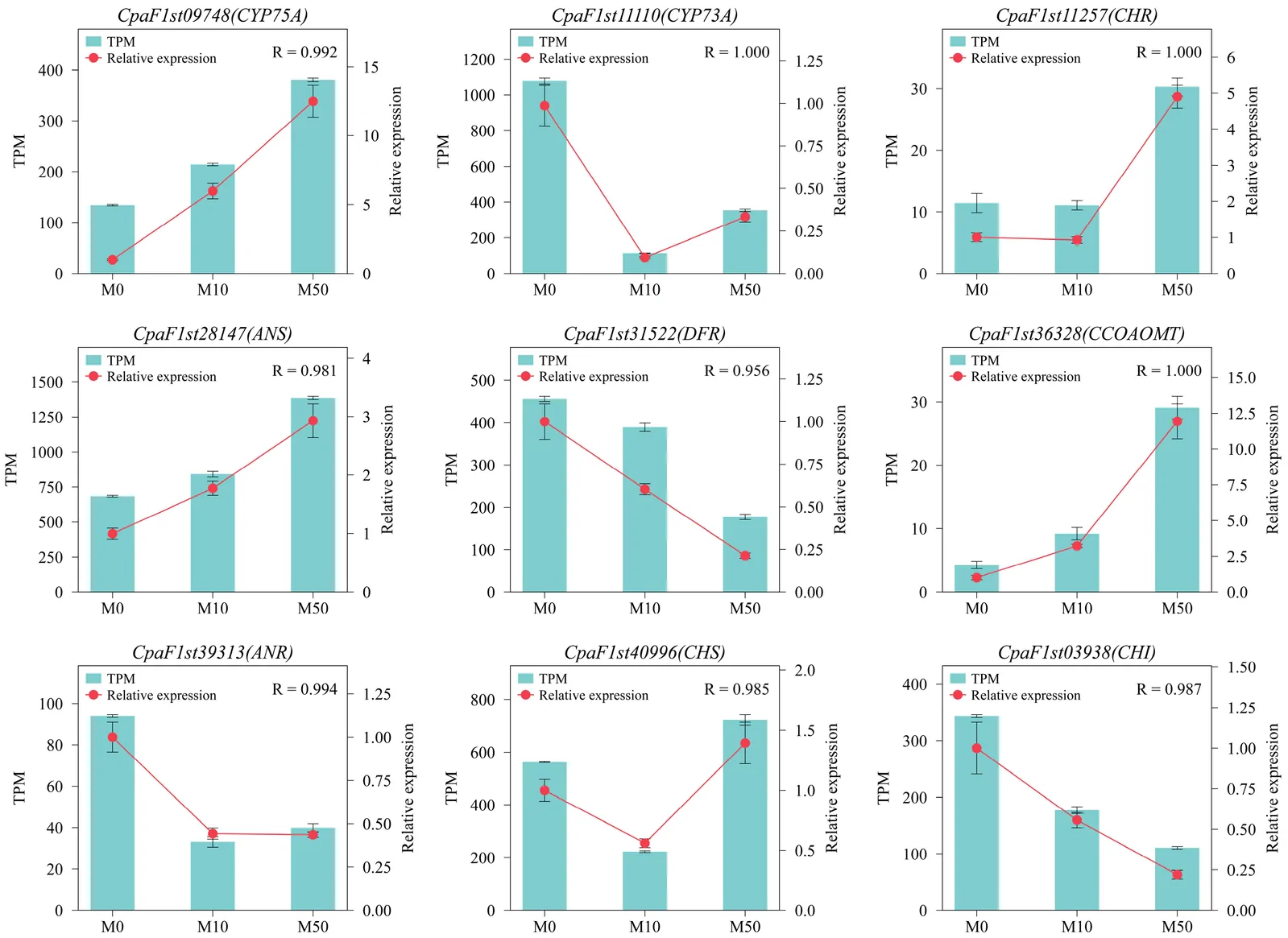
RT-qPCR validation of key flavonoid biosynthesis-related genes.

Reverse transcription quantitative real-time PCR (RT-qPCR) analysis confirmed that the expression patterns of key flavonoid biosynthesis-related genes were generally consistent with the transcriptomic data (Figure 7), with all R values exceeding 0.900, further validating the reliability of the transcriptomic results.

### 2.6. Gene-Metabolite Correlation Analysis Within the Flavonoid Biosynthesis Pathway

**Figure 8:**
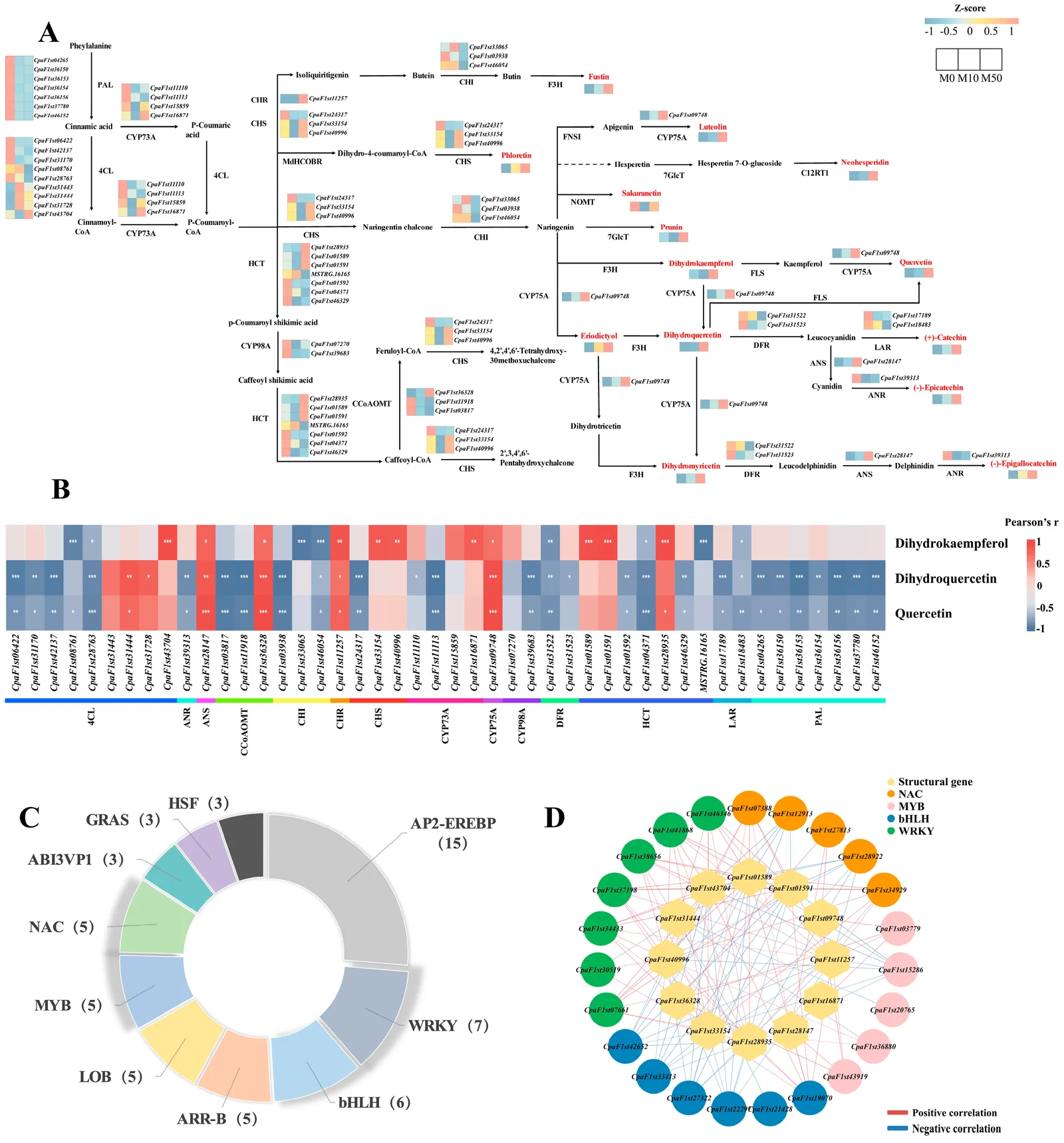
Transcriptome-metabolomic combined analysis. (**A**) Flavonoid biosynthetic pathway map. (**B**) Correlation heatmap between flavonoid-related differentially expressed genes and differentially accumulated metabolites. (**C**) Transcription factor annotation. (**D**) Co-expression network between transcription factors and flavonoid-related differentially expressed genes. **Note**: * *p* < 0.05; ** *p* < 0.01; *** *p* < 0.001.

To elucidate the regulatory mechanism by which MeJA affects flavonoid biosynthesis, transcriptomic and metabolomic data were integrated to construct a flavonoid biosynthesis pathway map (Figure 8A). For the correlation analysis, 46 flavonoid biosynthesis-related DEGs described above and three DAMs (dihydrokaempferol, dihydroquercetin, and quercetin) were selected as analytical targets. Pearson correlation coefficients (r) were calculated for each gene-metabolite pair, and a gene-metabolite correlation heatmap was generated (Figure 8B and Table S5). Dihydrokaempferol was positively and significantly correlated with several structural genes, including *CpHCT* (*CpaF1st01589*), *CpCYP73A* (*CpaF1st16871*), and *CpCHS* (*CpaF1st33154*), and negatively and significantly correlated with *CpCHI* (*CpaF1st33065* and *CpaF1st46054*) and *CpDFR* (*CpaF1st31522*). For dihydroquercetin, the positively and significantly correlated structural genes included *CpCCoAOMT* (*CpaF1st36328*), *CpCYP75A* (*CpaF1st09748*) and *CpANS* (*CpaF1st28147*), while *CpHCT* (*CpaF1st04371*), *CpCHI* (*CpaF1st03938*) and *CpCYP73A* (*CpaF1st11113*) were negatively and significantly correlated. Quercetin displayed a correlation pattern highly similar to that of dihydroquercetin. In total, 40 structural genes showed strong, statistically significant correlations with at least one of the three DAMs, including 12 positively and significantly correlated and 28 negatively and significantly correlated genes. These distinct gene sets suggest branch-specific regulatory patterns within the flavonoid biosynthetic pathway.

### 2.7. Identification of Candidate Transcription Factors Regulating Flavonoid Biosynthesis

Among 1144 DEGs shared by all three comparisons (M0 vs. M10, M0 vs. M50, and M10 vs. M50), 78 transcription factors (TFs) were identified, spanning 24 TF families. The number of DEGs assigned to each TF family was counted, and the top 10 TF families were selected for visualization as a circular plot (Figure 8C). The AP2-EREBP family contained the largest number of annotated DEGs (15), followed by the WRKY (7) and bHLH (6) families. The ARR-B, LOB, MYB, and NAC families each contained 5 DEGs and were among the most abundant TF families.

Based on the gene-metabolite correlation analysis, 12 structural genes positively correlated with dihydrokaempferol, dihydroquercetin, and quercetin were identified. These genes were integrated with the MYB, bHLH, NAC, and WRKY TFs annotated in this study, and a co-expression network was constructed. As shown in Figure 8D, *CpbHLH162* and *CpNAC073* showed the largest numbers of positively correlated genes. They were each positively and significantly associated with 8 structural genes, including *Cp4CL* (*CpaF1st43704*), *CpHCT* (*CpaF1st01589*, *CpaF1st01591*, *CpaF1st28935*), *CpCHR* (*CpaF1st11257*), *CpANS* (*CpaF1st28147*), and *CpCHS* (*CpaF1st33154*, *CpaF1st40996*) (Table S6). These results suggested that *CpbHLH162* and *CpNAC073* may act as candidate positive regulators of flavonoid biosynthesis in *C. paliurus* callus.

## 3. Discussion

### 3.1. MeJA-Induced Metabolomic Reprogramming and Flavonoid Accumulation

MeJA functions as a versatile elicitor that mimics environmental stress signals and remodels plant secondary metabolism, including the biosynthesis of flavonoids, phenolic acids and terpenoids [11,12,23,24]. In the present work, MeJA exerted prominent effects on flavonoid biosynthesis, as flavonoid biosynthesis pathways were significantly enriched across all three pairwise comparisons. The relative abundances of dihydrokaempferol, dihydroquercetin and quercetin, which represent key bioactive constituents in *C. paliurus*, increased markedly with rising MeJA concentrations. Likewise, the total flavonoid content showed a parallel increase. Such MeJA-stimulated flavonoid production has been documented in several plant species such as *Hedera helix* and *Carthamus tinctorius* [25,26]. Exogenous MeJA treatment elevated total flavonoid levels in *Hedera helix* and promoted anthocyanin deposition in safflower [25,26]. Nevertheless, metabolomic reprogramming triggered by MeJA exhibits inter-species variability. MeJA preferentially boosted triterpenoid biosynthesis in *Aralia elata* and *Eriobotrya japonica* [17,18]. These disparate metabolic outputs demonstrate that MeJA-initiated signaling does not drive a fixed metabolic cascade. Instead, downstream metabolic outcomes are shaped by species-specific pathway configurations and inherent biosynthetic preferences.

### 3.2. Coordinated Regulation of Flavonoid Structural Genes and Metabolite Accumulation

Structural genes encode catalytic enzymes that govern sequential reactions within flavonoid biosynthetic routes; their abundance largely dictates metabolic flux and final flavonoid production [27]. Flavonoid biosynthesis is highly conserved across plant taxa and originates from the phenylpropanoid pathway [28]. Within this upstream module, phenylalanine is converted into P-Coumaroyl-CoA via PAL, C4H and 4CL. Subsequent enzymatic reactions catalyzed by CHS, CHI, F3H, FLS and other enzymes partition metabolic intermediates toward distinct flavonoid sub-branches [29,30]. For example, in *Pyrus communis* callus, MeJA up-regulated early biosynthetic genes (*PcCHS*, *PcCHI*, *PcF3H*) together with late-stage genes (*PcDFR*, *PcANS*, *PcANR2a*, *PcLAR1*) to stimulate anthocyanin formation [31], while MeJA induced *AmCHS*, *AmF3H* and *AmFLS*, consequently raising kaempferol glycoside derivatives in *Astragalus membranaceus* [32]. In addition, in *Epimedium sagittatum*, MeJA application enhanced icariin (a prenylated flavonol glycoside) content through up-regulation of *PAL2*, *4CL1*, *4CL2*, *CHS2*, *CHI2*, *F3H* and *FLS1* [33]. In our study, *Cp4CL* (*CpaF1st43704*), *CpCHS* (*CpaF1st33154*, *CpaF1st40996*), *CpCYP73A* (*CpaF1st16871*), *CpHCT* (*CpaF1st01589*, *CpaF1st01591*, *CpaF1st28935*), *CpCCoAOMT* (*CpaF1st36328*), *CpCYP75A* (*CpaF1st09748*), *CpANS* (*CpaF1st28147*) and *CpCHR* (*CpaF1st11257*) displayed significant positive correlations with at least one of the three DAMs: dihydrokaempferol, dihydroquercetin or quercetin. In contrast, 28 flavonoid biosynthesis genes were significantly negatively correlated with at least one of these three metabolites. This observation is attributed to the fact that flavonoid metabolism forms a branched biosynthetic network rather than a simple linear pathway [34,35]. First, a time lag exists between gene transcription, enzyme activity and metabolite accumulation [36]. Gene expression only captures the instantaneous transcriptional state at sampling. While gene transcription peaked at M10, pre-synthesized enzymes remained active at higher MeJA concentrations, and flavonoids stably stored in vacuoles support sustained metabolite accumulation. Second, redistribution of metabolic flux among flavonoid sub-branches occurs under different MeJA concentrations [37]. M10 is optimal for inducing a subset of biosynthetic genes; as concentration rises, transcription of these genes declines while genes of other flavonoid branches are activated, sustaining the increase in total flavonoids. Third, MeJA exerts concentration-dependent regulation [37]. Low-to-moderate MeJA induces specific biosynthetic genes. M10 triggers feedback regulation of partial genes, whereas higher concentrations remodel the jasmonate signaling pathway and redirect metabolic flux via distinct transcription factors.

### 3.3. CpbHLH162 and CpNAC073 as Candidate Hub Regulators of Flavonoid Biosynthesis

TFs act as critical intermediates bridging MeJA signaling cascades and flavonoid structural genes. By recognizing cis-regulatory motifs in target gene promoters, TFs synergistically stimulate structural gene expression and facilitate flavonoid biosynthesis [38,39]. TF annotation revealed 78 shared differentially expressed TFs among the three MeJA-treated groups, which were assigned to 24 TF families. Among them, WRKY, bHLH, MYB and NAC families are well-established modulators of plant secondary metabolism [40,41]. Further co-expression network analysis between TFs and structural genes pinpointed *CpbHLH162* and *CpNAC073* as potential positive regulators. Both TFs showed strong positive correlations with 8 structural genes (*Cp4CL*, *CpCHS*, *CpCHR*, *CpHCT* and *CpANS*). Functional studies from other plant species support the central regulatory roles of bHLH and NAC family members. For example, in *Brassica campestris*, MeJA-responsive *bHLH42* accelerated anthocyanin accumulation by positively modulating *CHI1* and *F3H* [42]. Additionally, *DoMYC2* concurrently activated *DoCHS8*, *DoF3H* and *DoANS* to trigger anthocyanin enrichment in *Dendrobium officinale* [43]. Furthermore, *CsNAC086* elevated flavonol production by inducing *CsFLS* expression in *Camellia sinensis* [44]. These published results are consistent with our data, confirming that MeJA promotes the biosynthesis of bioactive compounds by activating TFs that regulate the expression of biosynthetic pathway genes.

### 3.4. Limitations of the Study

Despite the insights gained from the present work, several inherent limitations should be acknowledged: (1) In this study, the aluminium trichloride (AlCl_3_) colorimetric method was used to determine total flavonoid content. From a chemical perspective, some non-flavonoid phenolic compounds, such as phenolic acids with vicinal dihydroxy groups, can still form colored complexes with Al^3+^. Such interference increases absorbance values and may lead to an overestimation of total flavonoid content. However, this method was adequate in this study, as it was used only for the relative comparison of flavonoid levels among different treatments. (2) *CpbHLH162* and *CpNAC073* were identified as hub regulators of flavonoid biosynthesis, making them promising candidates for further functional characterization and metabolic engineering. However, the correlative nature of our data precludes definitive causal conclusions, and whether these TFs regulate the seven target structural genes directly or indirectly, and whether they act cooperatively, requires experimental validation. Future studies integrating yeast one-hybrid, dual-luciferase reporter, and transient overexpression or gene silencing approaches will be essential to verify the proposed regulatory network and to unlock the potential of MeJA-mediated flavonoid enhancement in *C. paliurus*. (3) It should be noted that callus rather than intact plants was used in this study. Callus is a dedifferentiated cell mass without tissue and organ structures. Compared with intact plants, the callus system lacks tissue-specific compartmentation and long-distance signal transduction. Therefore, the metabolic and transcriptional responses observed in *C. paliurus* callus may not fully recapitulate the regulatory characteristics of flavonoid biosynthesis in whole plants. In intact plants, MeJA signaling can be transported across tissues, and flavonoid synthesis and accumulation exhibit tissue-specific patterns in leaves, stems and roots. Such spatial regulatory features are absent in undifferentiated callus cells. In addition, intact plants possess mature defense networks and complex hormone crosstalk, which may alter the sensitivity to MeJA and the final flavonoid yield relative to in vitro callus. Hence, the MeJA concentration-dependent flavonoid regulatory pattern obtained from callus in this study represents a cellular-level response. The response magnitude and gene expression trends may show discrepancies when extrapolated to whole plants.

## 4. Materials and Methods

### 4.1. Plant Materials and Callus Culture

The experimental material was friable, undifferentiated leaf-derived callus of *C. paliurus*, subcultured for 15 generations (20 days per subculture cycle). The original leaves were collected from a 2-year-old potted seedling numbered 111. This plant was grown from seed collected in Wufeng Tujia Autonomous County (30°12′ N, 110°40′ E), Yichang City, Hubei Province, China. This plant was an excellent individual previously selected by our research team for its high content of bioactive compounds. The experiment used MS as the basal medium, supplemented with 0.2 mg/L KT + 0.5 mg/L 6-BA + 0.75 mg/L NAA + 0.4 mg/L 2,4-D, along with 30 g/L sucrose and 6.5 g/L agar; the pH was adjusted to 5.8. All cultures were maintained in the dark at 23 ± 2 °C.

### 4.2. MeJA Treatment and Sampling

During the medium preparation, MeJA solutions at final concentrations of 10 μM (M10) and 50 μM (M50) were filter-sterilized and incorporated into the above medium ingredients. At the 15th subculture, the callus was immediately inoculated onto the MeJA-containing medium and the medium was not refreshed during the subsequent culture period. Based on the callus subculture cycle, the MeJA treatment period was set to 20 days. Each treatment had three biological replicates, with 20 flasks per replicate. Each flask was inoculated with three pieces of uniformly growing callus at approximately 0.3 g fresh weight. For biomass determination, at each sampling time point (5, 10, 15, and 20 days), each treatment had three replicates. For each replicate, one flask of callus was collected (3 flasks per treatment per time point). The callus was carefully separated from the medium, freed of residual medium, blotted dry on filter paper, and weighed to record the fresh weight. The callus was then oven-dried at 60 °C to a constant weight, and the dry weight was recorded. In addition, after 20 days of culture, each treatment had three replicates. For each replicate, three flasks of callus were collected (9 flasks per treatment), and the callus was snap-frozen in liquid nitrogen. All samples were sent to Gene Denovo Biotechnology Co., Ltd. (Guangzhou, China) for transcriptomic sequencing and untargeted metabolomic analysis.

Preparation of the MeJA solution: MeJA (CAS: 39924-52-2; molecular weight: 224.3) was purchased from Macklin Biochemical Technology Co., Ltd. (Shanghai, China). A 100 mmol/L stock solution was prepared by dissolving 1.1215 g MeJA in anhydrous ethanol and making up the volume to 50 mL. The solution was then filter-sterilized through a 0.22 μm microporous membrane and stored at −20 °C in the dark until use.

### 4.3. Untargeted Metabolomic Analysis

The samples were ground to a fine powder in liquid nitrogen, and approximately 100 mg of each powdered sample was weighed. Quality control (QC) samples were prepared by mixing equal aliquots of all samples. The powder was extracted with 1 mL of pre-cooled methanol/acetonitrile/water (2:2:1, *v*/*v*/*v*), vortexed, and sonicated twice at low temperature for 30 min each time. The mixture was then incubated at −20 °C for 60 min and centrifuged at 14,000× *g* for 20 min at 4 °C. The supernatant (equivalent to 50 mg of sample) was collected and dried under vacuum. The dried residue was reconstituted in 100 μL of acetonitrile/water (1:1, *v*/*v*), vortexed, and centrifuged at 14,000× *g* for 15 min at 4 °C. The resulting supernatant was used for UHPLC-MS/MS analysis and each treatment was performed in three biological replicates.

Chromatographic separation was performed on a 2.1 mm × 100 mm ACQUITY UPLC BEH Amide 1.7 μm column (Waters, Wexford, Ireland) using a UHPLC system (1290 Infinity LC, Agilent Technologies, Santa Clara, CA, USA) coupled to an electrospray ionization (ESI) quadrupole time-of-flight mass spectrometer (AB SCIEX TripleTOF 6600, AB Sciex LLC, Framingham, MA, USA). In both ESI positive and negative modes, the mobile phase contained A = 25 mM ammonium acetate and 25 mM ammonium hydroxide in water and B = acetonitrile. The gradient was 85% B for 1 min and was linearly reduced to 65% in 11 min, further decreased to 40% in 0.1 min and kept for 4 min, and finally increased to 85% in 0.1 min, followed by a 5 min re-equilibration period. To control systematic bias and instrumental drift during MS acquisition, pooled QC samples were injected every ten injections throughout the entire acquisition sequence. The stability of pooled QC samples was used to confirm reliable instrument performance during the analytical run. Solvent blank controls were incorporated into the UHPLC-MS/MS injection sequence. The ion features present in solvent blanks were filtered out from the feature matrix to remove background noise and interference.

The raw data were converted to MzXML files using ProteoWizard MSConvert (v3.0.6428) before importing into freely available XCMS software (v3.7.1). For peak picking, the following parameters were used: centWave *m*/*z* = 10 ppm, peakwidth = c (10, 60), prefilter = c (10, 100). For peak grouping, bw = 5, mzwid = 0.025, minfrac = 0.5 were used. CAMERA package was used for the annotation of isotopes and adducts. In the extracted ion features, only the variables having more than 50% of the nonzero measurement values in at least one group were kept. Compound identification of metabolites was performed by comparing the accurate *m*/*z* values (<10 ppm) and MS/MS spectra against an in-house database established with available authentic standards, together with public databases including MassBank, Metlin, and MassBank of North America (MoNA) to assist metabolite annotation. The missing data were filled using the K-Nearest Neighbor (KNN) method, and features with relative standard deviation (RSD) greater than 50% were filtered out.

OPLS-DA was performed using the ropls package (v1.43.0) in R software (v4.2.1). The OPLS-DA model was validated by cross-validation and a 200-time permutation test was performed to prevent model overfitting. According to previous studies [45,46,47], DAMs were identified based on variable importance in projection (VIP) values from the OPLS-DA models, *p* values, and fold change (FC) values, with thresholds of VIP ≥ 1, *p* < 0.05, and |log2FC| > 1. Multiple testing correction using the Benjamini–Hochberg FDR method was not applied. Raw *p*-values were reported for exploratory screening of differential features in untargeted metabolomics. Low-quality features were pre-filtered to reduce noise before statistical analysis. The relative contents of the DAMs were subjected to Z-score normalization using the pheatmap package (v1.0.12) in R software and cluster heatmaps were generated. KEGG enrichment results were visualized using the Omicshare platform (https://www.omicshare.com).

### 4.4. Determination of Total Flavonoid Content

The callus samples returned after metabolomic and transcriptomic analysis were oven-dried and ground into powder using a ball mill. The resulting powder was used for total flavonoid content determination, and the test solution was prepared according to Cao et al. [48]. Briefly, 0.4 g of sample was placed in a 20 mL stoppered test tube, and 5 mL of 70% (*v*/*v*) ethanol was added. After vortexing for 1 min, the mixture was ultrasonically extracted for 45 min and then heated in a water bath at 70 °C for 45 min. After cooling, the supernatant was transferred to a 10 mL centrifuge tube and centrifuged at 10,000 rpm for 5 min at 4 °C, and the resulting supernatant was collected as the test solution. Total flavonoid content was determined using the aluminum trichloride colorimetric method following Li et al. [49]. A 0.1 mL aliquot of the sample solution was pipetted into a 10 mL centrifuge tube, mixed with 1 mL of 1% (*w*/*v*) aluminum trichloride in methanol, and diluted to 10 mL with methanol. In this method, rutin was used as the standard, absorbance was measured at 410 nm, and the calibration equation was A = 0.02842C + 0.00097 with R^2^ = 0.9997 and a linear range of 0–275 μg. The method showed good precision (RSD = 0.669%), reproducibility (RSD = 0.991%), and stability, with the colored complex remaining stable for 90 min. The mean spike recovery was 100.186%, with an RSD of 1.227%. Total flavonoid content was calculated from a rutin standard curve and expressed on a dry-weight (DW) basis. These dry-weight values were then converted to fresh-weight (FW) values using the following equation:

$${\mathrm{TF}}_{\mathrm{FW}}\;=\;{\mathrm{TF}}_{\mathrm{DW}}\;\times \;\frac{\mathrm{DW}}{\mathrm{FW}}$$

where TF_FW_ is the total flavonoid content on a fresh-weight basis (mg/g FW); TF_DW_ is the total flavonoid content on a dry-weight basis (mg/g DW); and DW/FW is the dry-to-fresh weight ratio.

### 4.5. Transcriptomic Sequencing

Total RNA was extracted using TRIzol™ reagent (Life Technologies, Carlsbad, CA, USA). RNA purity and concentration were determined using a NanoDrop™ 2000 spectrophotometer (Thermo Fisher Scientific, Waltham, MA, USA), and RNA integrity was assessed with an Agilent 2100 Bioanalyzer (Agilent Technologies, Santa Clara, CA, USA). RNA samples with an RNA integrity number (RIN) ≥ 7.0 were selected for cDNA library construction. The libraries were sequenced on the Illumina NovaSeq X Plus platform (Illumina, San Diego, CA, USA) in paired-end mode with a read length of 2 × 150 bp (PE150). Raw reads were quality-filtered using fastp (v0.18.0) to obtain clean reads, which were aligned to the *C. paliurus* reference genome using HISAT2 (v2.1.0). Based on the alignment results, StringTie was used for transcript reconstruction, and gene expression levels were quantified as transcripts per million (TPM) values using RSEM (v1.2.19). The expected read counts generated by RSEM were used as the input for differential expression analysis, which was performed among the M0, M10, and M50 treatments using the DESeq2 package (v1.20.0) in R software. Genes with |log2FC| > 1 and FDR < 0.05 were identified as DEGs. The TPM values of the DEGs were Z-score-normalized using the pheatmap package (v1.0.12) in R software and cluster heatmaps were generated. GO and KEGG enrichment analyses were performed using the Omicshare platform (https://www.omicshare.com).

### 4.6. RT-qPCR Analysis

RT-qPCR was performed to validate the expression levels of the 9 candidate genes. Total RNA was reverse-transcribed into first-strand cDNA using a HiScript III 1st Strand cDNA Synthesis Kit (+gDNA wiper) (Vazyme Biotech Co., Ltd., Nanjing, China) following the manufacturer’s instructions. RT-qPCR was conducted on a Bio-Rad CFX96 Real-Time PCR platform (Bio-Rad, Hercules, CA, USA). The specific program parameters were set according to the manufacturer’s instructions for Taq Pro Universal SYBR qPCR Master Mix (Vazyme Biotechnology Co., Nanjing, China). The *C. paliurus* 18S rRNA gene was used as the reference gene [50]. Gene-specific primers were designed based on the gene sequences using Primer Premier 5.0, and the primer information is listed in Table S7. Relative expression levels were calculated using the 2^−ΔΔCt^ method. All experiments were performed in three biological replicates. No-template negative controls (NTC) were included in each qPCR run to detect potential reagent or primer contamination.

### 4.7. Statistical Analysis

All data were presented as the mean ± standard deviation (SD). Statistical analyses were performed using Microsoft Excel 2021 and IBM SPSS Statistics 29.0. Differences among treatments were assessed by one-way analysis of variance (ANOVA) followed by Duncan’s multiple range test, and statistical significance was set at *p* < 0.05.

## 5. Conclusions

Combined metabolomic and transcriptomic analysis revealed the molecular responses of *C. paliurus* callus to MeJA elicitation. Exogenous MeJA induced obvious metabolic and transcriptional reprogramming and enhanced the accumulation of dihydrokaempferol, dihydroquercetin and quercetin. Twelve key structural genes involved in flavonoid biosynthesis (two *4CL* genes, two *CHS* genes, one *CYP73A* gene, three *HCT* genes, one *CCoAOMT* gene, one *CHR* gene, one *CYP75A* gene, and one *ANS* gene) were co-upregulated. Two hub TFs, *CpbHLH162* and *CpNAC073*, were identified as potential upstream regulators of flavonoid biosynthesis. This study provides candidate genes for metabolic improvement of *C. paliurus* callus. Since the conclusions are derived from correlative omics data, subsequent molecular biology experiments are necessary to validate the proposed regulatory module.

## Data Availability

The raw RNA sequencing data generated in this study have been deposited in the Genome Sequence Archive (GSA) of the National Genomics Data Center (NGDC, http://ngdc.cncb.ac.cn/) under BioProject accession number PRJCA073555. Other data supporting the findings are included in the article and its Supplementary Materials. Further inquiries can be directed to the corresponding author.

## Supplementary Materials

The following supporting information can be downloaded at: https://www.mdpi.com/article/10.3390/plants15203135/s1, Table S1: Biomass analysis; Table S2: All DAMs which were identified; Table S3: Transcriptomic sequencing data quality; Table S4: All DEGs which were identified; Table S5: Correlations between flavonoid-related DEGs and DAMs; Table S6: Correlations between candidate TFs and flavonoid-related genes; Table S7: Primer sequences used for RT-qPCR.
